# Supplementary figures and images for: A genetic screen in Drosophila uncovers the multifaceted properties of the NUP98-HOXA9 oncogene
Source: PLoS Genet. 2021 Aug 12;17(8):e1009730. doi: 10.1371/journal.pgen.1009730 (PMC8384169; doi:10.1371/journal.pgen.1009730)

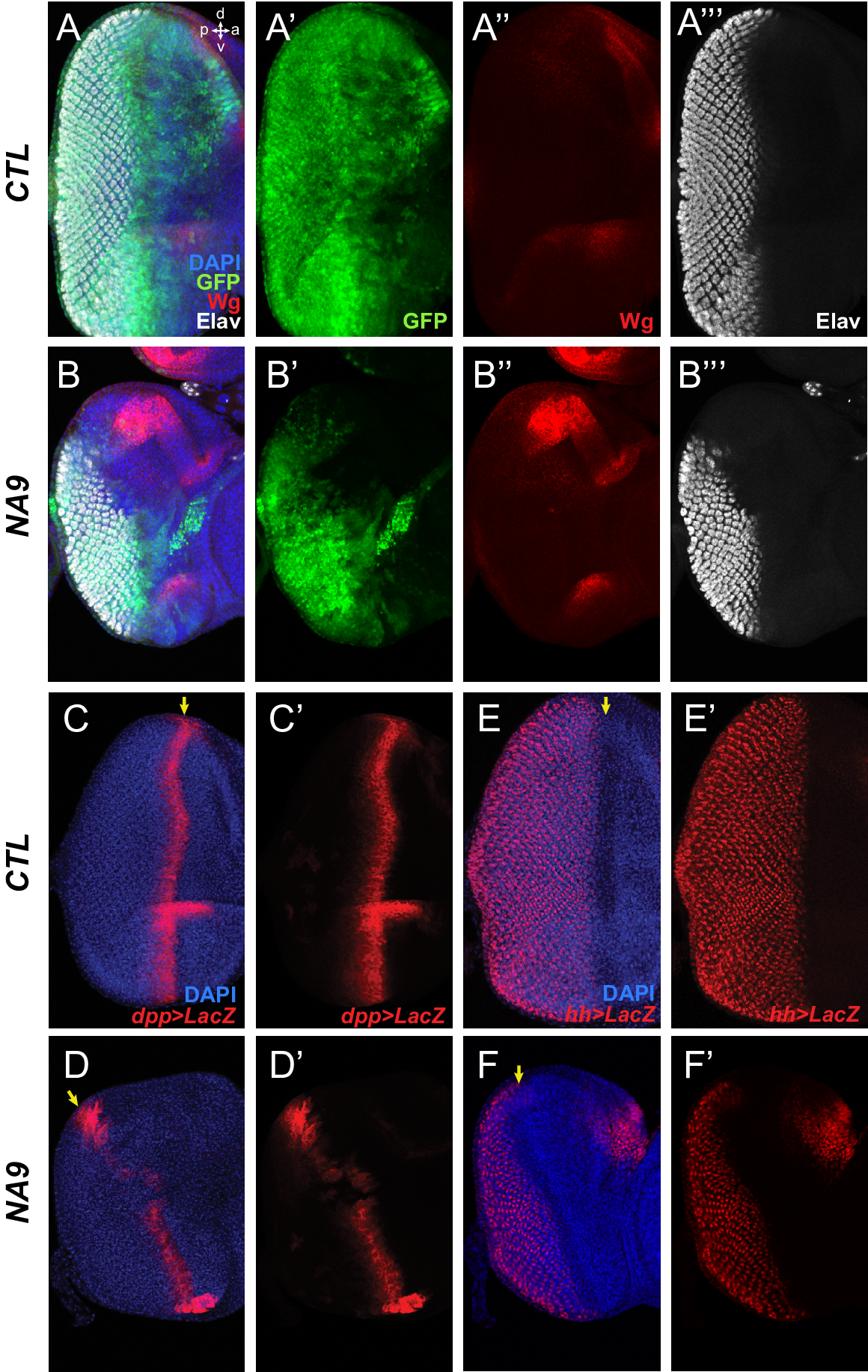

Supplement: S1 Fig — Third instar larval eye discs immunostained with (A, B) anti-Wingless (Wg) and anti-Elav (marks photoreceptor neurons). (C, D) Anti-βGal stainings (red) report on dpp expression. (E, F) Anti-βGal stainings (red) report on hh expression. DAPI stainings mark the nuclei. GFP delineates the areas of ey-Gal4 activity. The following genotypes were analyzed: (A) ey-Gal4/+; UAS-GFP.nls/+ referred to as CTL. (B) ey-Gal4/UAS-NA9; UAS-GFP.nls/+ referred to as NA9. (C) ey-Gal4/dpp-LacZ referred to as CTL. (D) ey-Gal4, UAS-NA9/dpp-LacZ referred to as NA9. (E) ey-Gal4/+; hh-LacZ/+ referred to as CTL. (F) ey-Gal4, UAS-NA9/+; hh-LacZ/+ referred to as NA9. (TIF) [file pgen.1009730.s001.tif]

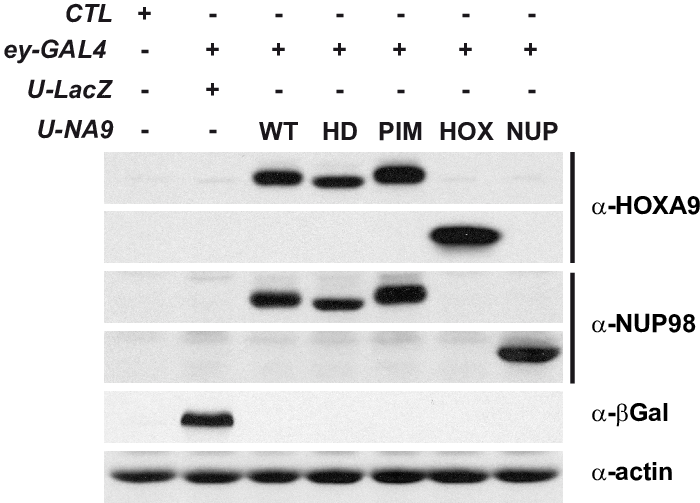

Supplement: S2 Fig — Immunoblots monitoring protein levels from whole larval extracts for the different NA9 variants used in this study. The ey-Gal4 line was used to drive the expression of UAS-LacZ or the following UAS constructs: UAS-NA9 (WT), UAS-NA9HD (HD), UAS-NA9PIM (PIM), UAS-HOXA9ΔNT (HOX) or UAS-NUP98ΔCT (NUP). Protein levels were assessed using antibodies against HOXA9 or NUP98. βGal levels were determined as control for ey-GAL4 activity. Actin levels were used as loading control. CTL corresponds to cell extracts made from the control w1118 line. (TIF) [file pgen.1009730.s002.tif]

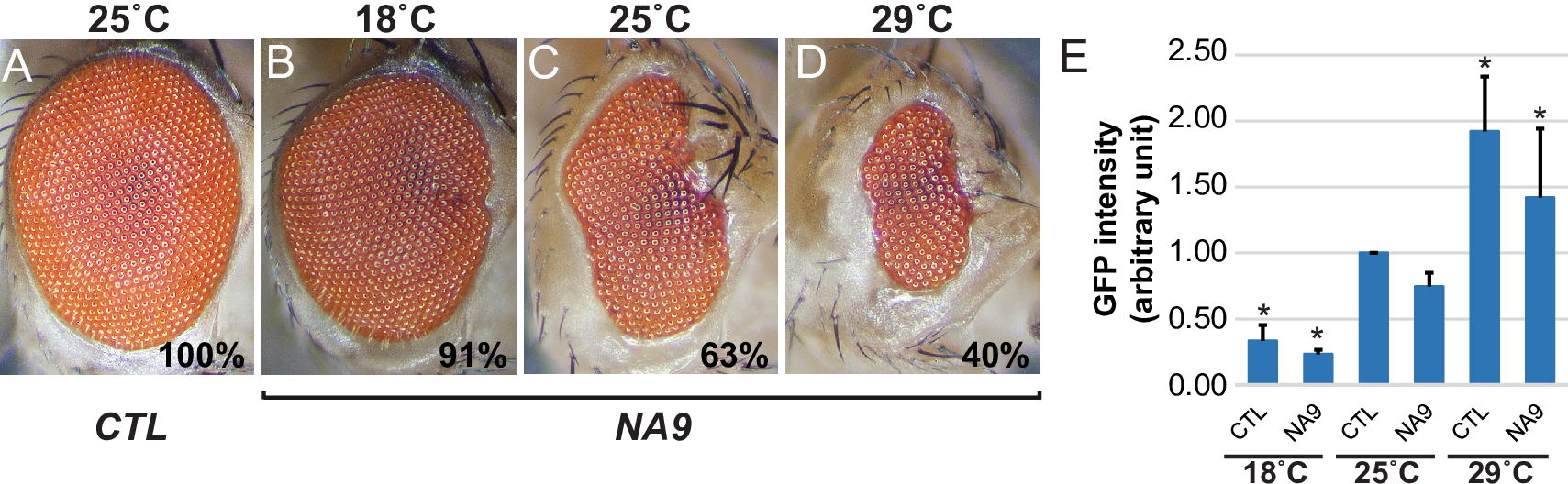

Supplement: S3 Fig — (A-D) Micrographs of adult Drosophila eyes of the following genotypes: (A) ey-Gal4/+; UAS-GFP/+ referred to as CTL. (B-D) ey-Gal4, UAS-NA9/+; UAS-GFP/+ referred to as NA9. Flies were raised at 18°C, 25°C or 29°C as indicated. Mean eye size (expressed as percent compared to CTL), is indicated at the bottom right of each eye micrograph. Five flies were quantified per condition. (E) Quantification of GFP fluorescence in eye imaginal discs. Expression of the NA9 transgene is enhanced by temperature elevation during development, which correlates with enhanced phenotypic strength in adult eyes. Stars denote statistically significant variations (p≤0.05, Student’s t-test) in GFP fluorescence compared to conditions at 25°C. (TIF) [file pgen.1009730.s003.tif]

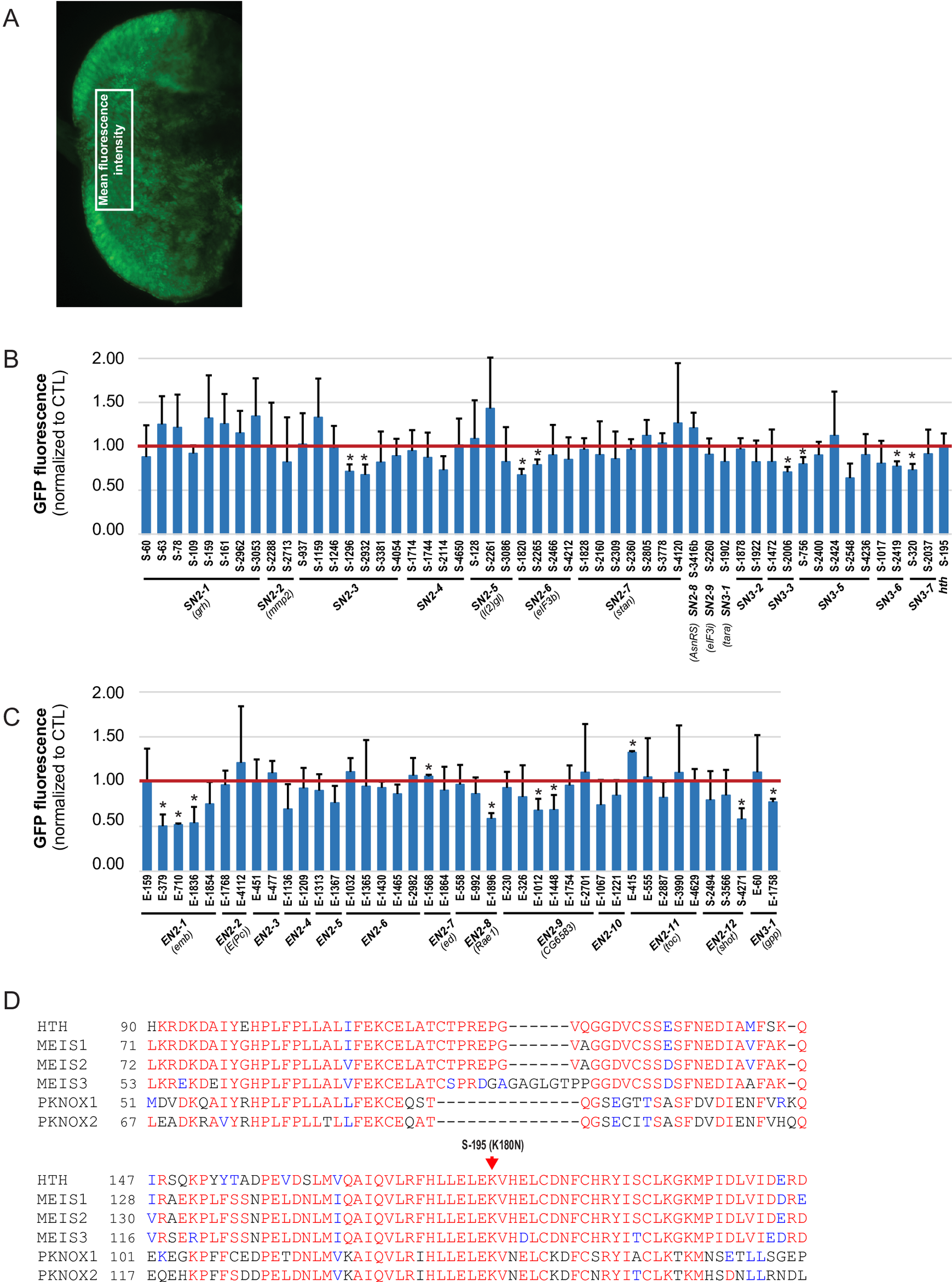

Supplement: S4 Fig — (A) Third instar eye imaginal disc expressing UAS-GFP.nls under ey-Gal4. Mean GFP fluorescence intensity was used as a proxy for assessing variation in ey-Gal4 activity. GFP fluorescence was quantified in the posterior region of eye discs (white box) using the Photoshop Measurement feature. The following genotypes were analyzed: (B) ey-Gal4/+; UAS-GFP.nls/suppressor alleles and (C) ey-Gal4/+; UAS-GFP.nls/enhancer alleles as indicated on the panels. Fluorescence intensities are normalized to CTL (ey-Gal4/+; UAS-GFP.nls/+). Error bars represent standard deviations (SD) from at least three independent experiments. The green and red stars highlight a statistically significant (p≤0.05, Student’s t-test) increase or decrease in GFP fluorescence, respectively. (D) Amino acid sequence alignment of PBX-interacting regions from Drosophila HTH and human MEIS-related homeobox proteins. Identical amino acids are shown in red, whereas similar amino acids are shown in blue. The position of the amino acid change (K180N) found in the hthS-195 allele is shown on top of the HTH sequence. (TIF) [file pgen.1009730.s004.tif]

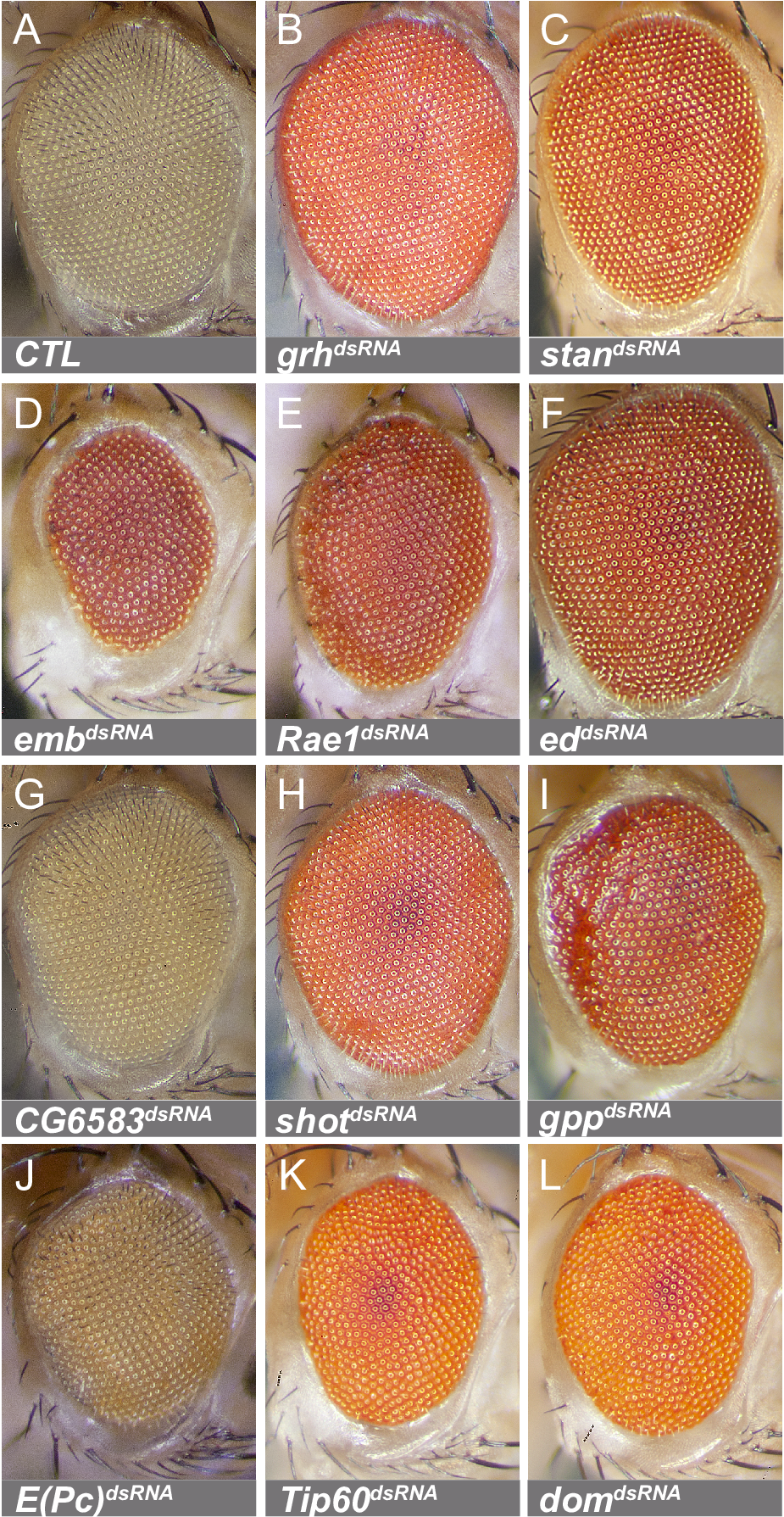

Supplement: S5 Fig — (A-L) Micrographs of representative adult Drosophila eyes of (A) ey-Gal4/+ referred to as CTL or (B-L) ey-Gal4/specific UAS-dsRNA constructs as indicated on the panels. (TIF) [file pgen.1009730.s005.tif]

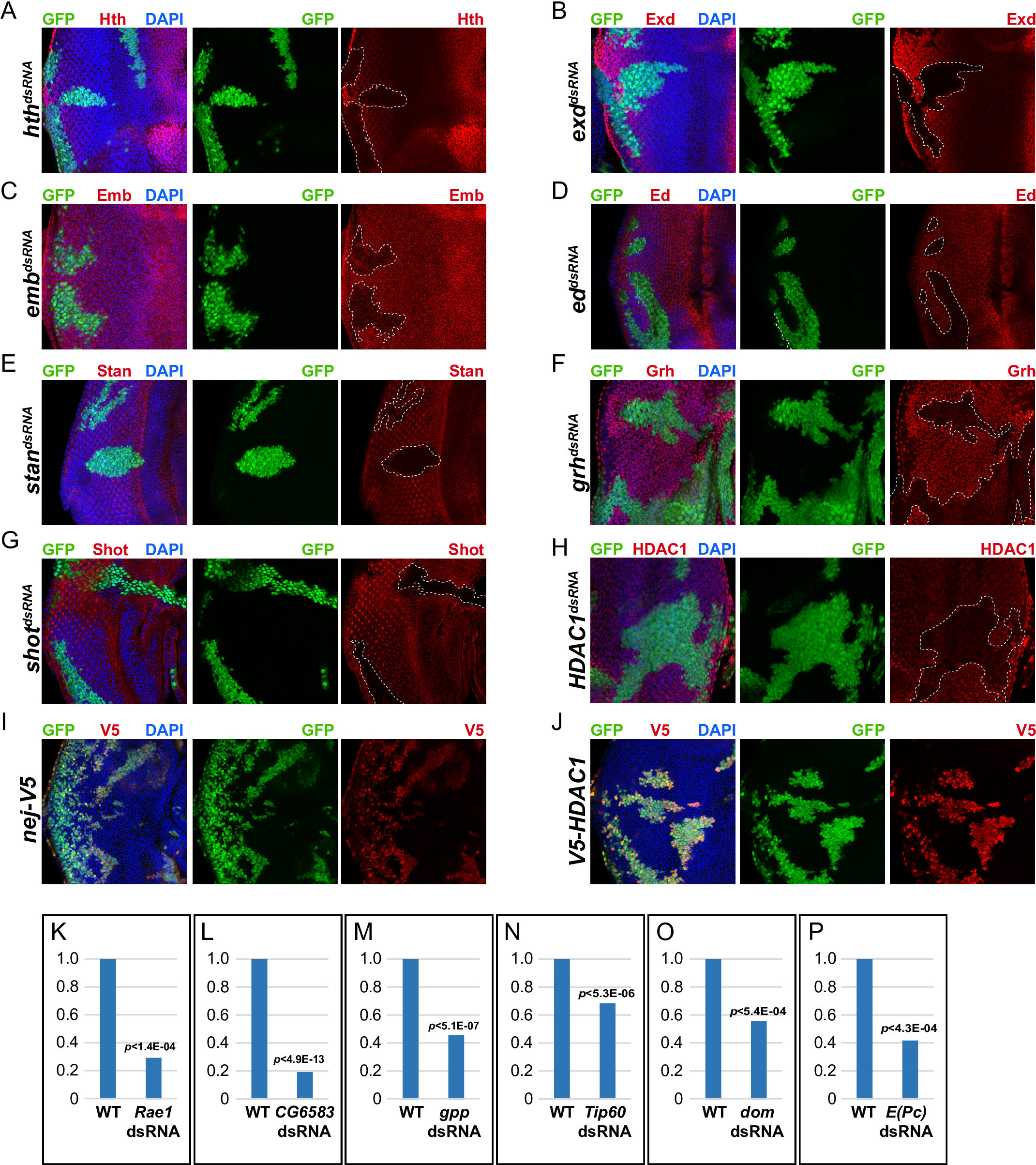

Supplement: S6 Fig — (A-J) Third instar larval eye discs were immunostained with the indicated antibodies (red) to monitor knockdown efficiency of (A-H) dsRNA lines or expression of (I, J) V5-tagged cDNAs as indicated to the left of each panel. DAPI staining marks the nuclei, whereas GFP fluorescence identifies the areas of transgene expression. The flp-out line (hs-flp; Act5C > CD8 > GAL4, UAS-GFP) was used to clonally induce the expression of GFP and the indicated UAS constructs as single copies. When antibodies were not available to assess knockdown efficiency, qPCR analysis were performed instead using mRNA transcripts isolated from eye discs (K-O) or lymph glands (P). The mean RQ values of at least two independent experiments are shown. Statistical significance was determined using a Student’s t-test. (TIF) [file pgen.1009730.s006.tif]

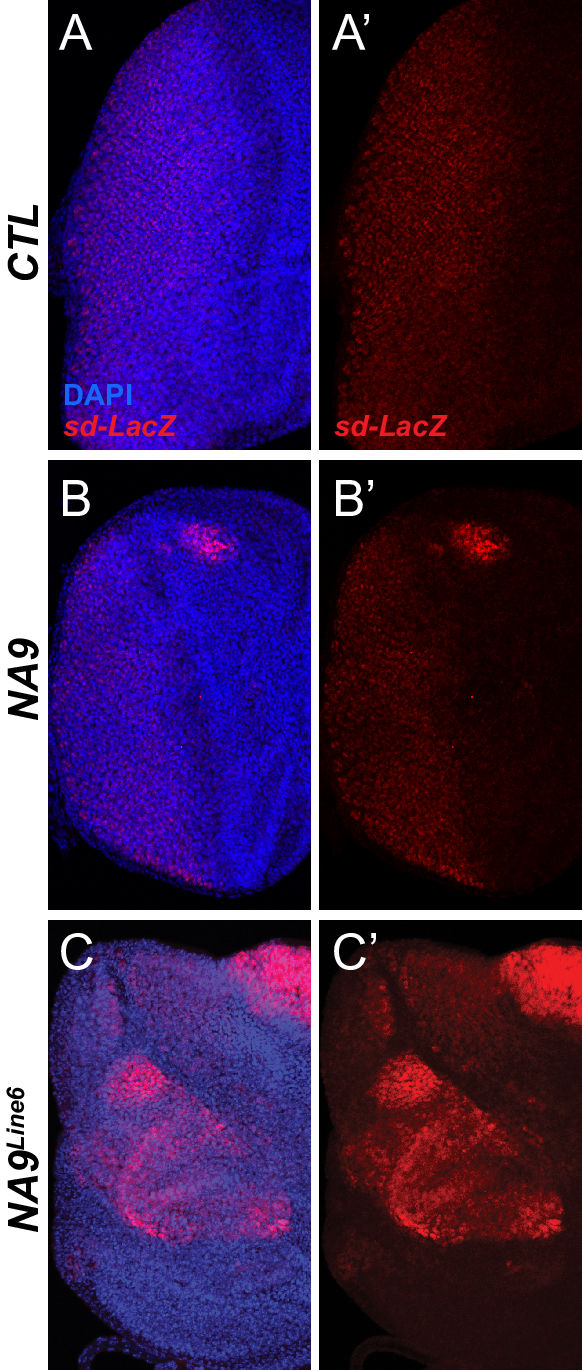

Supplement: S7 Fig — Third instar larval eye discs immunostained with anti-βGal as a reporter for sd-LacZ expression. The following genotypes were analyzed: (A) sd-LacZ/+; ey-Gal4/+ referred to as CTL. (B) sd-LacZ/+; ey-Gal4/UAS-NA9 referred to as NA9. (C) sd-LacZ/+; ey-Gal4/UAS-NA9line6 referred to as NA9line6. DAPI staining marks the nuclei. (TIF) [file pgen.1009730.s007.tif]

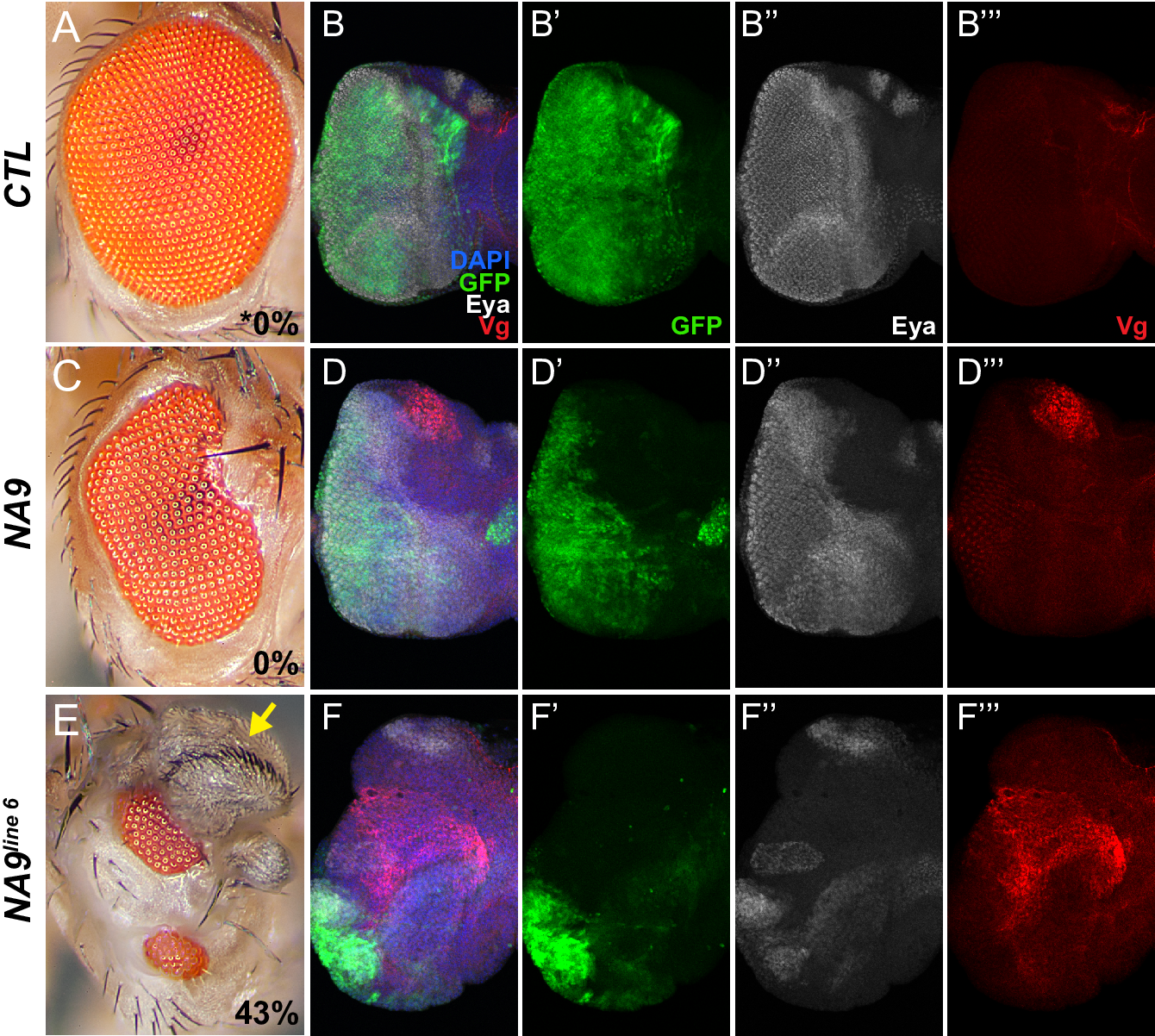

Supplement: S8 Fig — (A, C, E) Micrographs of adult Drosophila eyes. (B, D, F) Third instar larval eye discs immunostained with anti-Vestigial (Vg) and anti-Eyes Absent (Eya) antibodies. DAPI staining marks the nuclei, whereas GFP defines the areas of transgene expression. The following genotypes were analyzed: (A, B) ey-Gal4/+; UAS-GFP.nls/+ referred to as CTL. (C, D) ey-Gal4/UAS-NA9; UAS-GFP.nls/+ referred to as NA9. (E, F) ey-Gal4/+; UAS-GFP.nls/UAS-NA9line6 referred to as NA9line6. The proportion (%) of eyes presenting ectopic wing formation is indicated at the bottom right of adult eye micrographs. Quantifications are shown in Table 2. Transgene expression of UAS-NA9line6 is approximately 6-fold higher compared to the UAS-NA9 used in this study and previously referred to as line 5 [35]. (TIF) [file pgen.1009730.s008.tif]

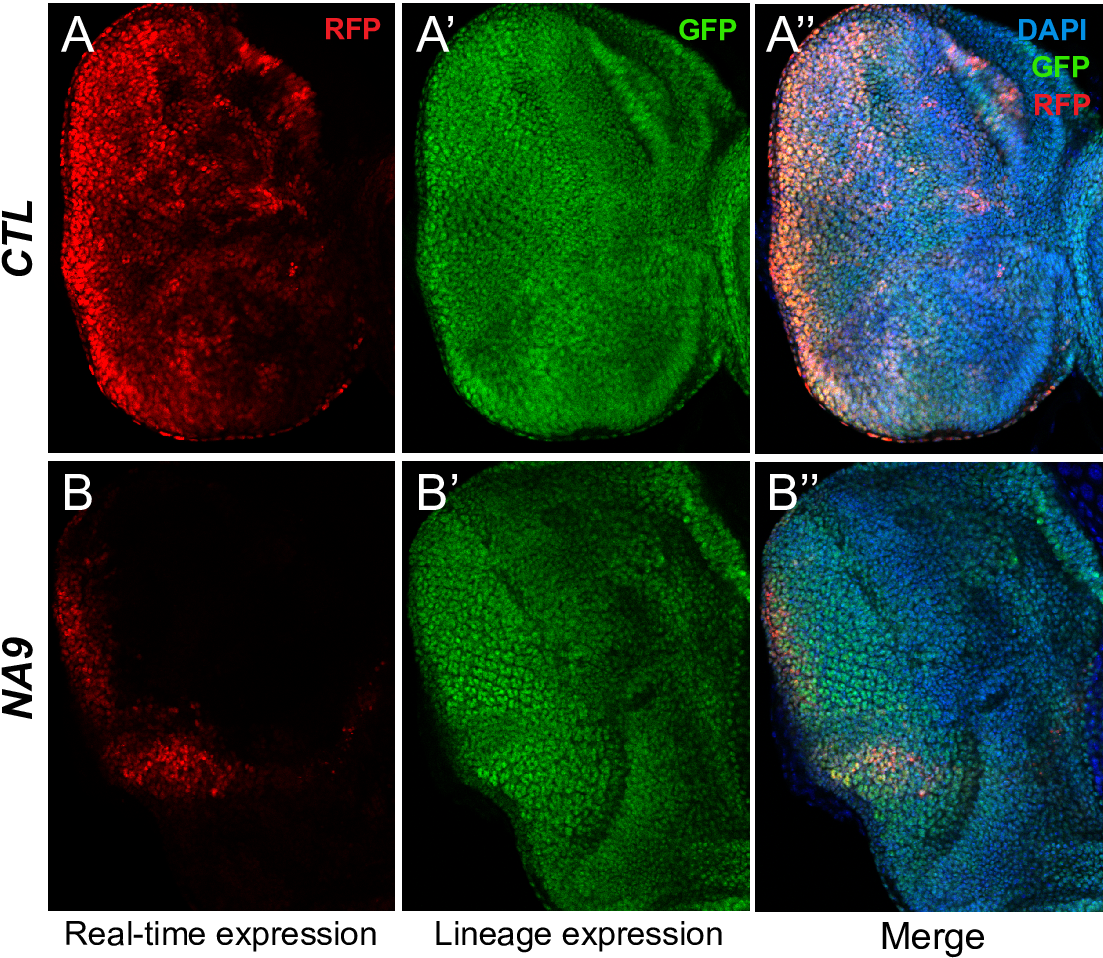

Supplement: S9 Fig — Real-time expression (RFP) and lineage expression (GFP) was determined for the following genotypes: (A) ey-Gal4/+; G-Trace/+ referred to as CTL. (B) ey-Gal4, NA9, G-Trace referred to as NA9. DAPI staining marks the nuclei. G-Trace refers to UAS-RFP, UAS-FLP, Ubi-p63E(FRT.STOP)nEGFP [60]. (TIF) [file pgen.1009730.s009.tif]

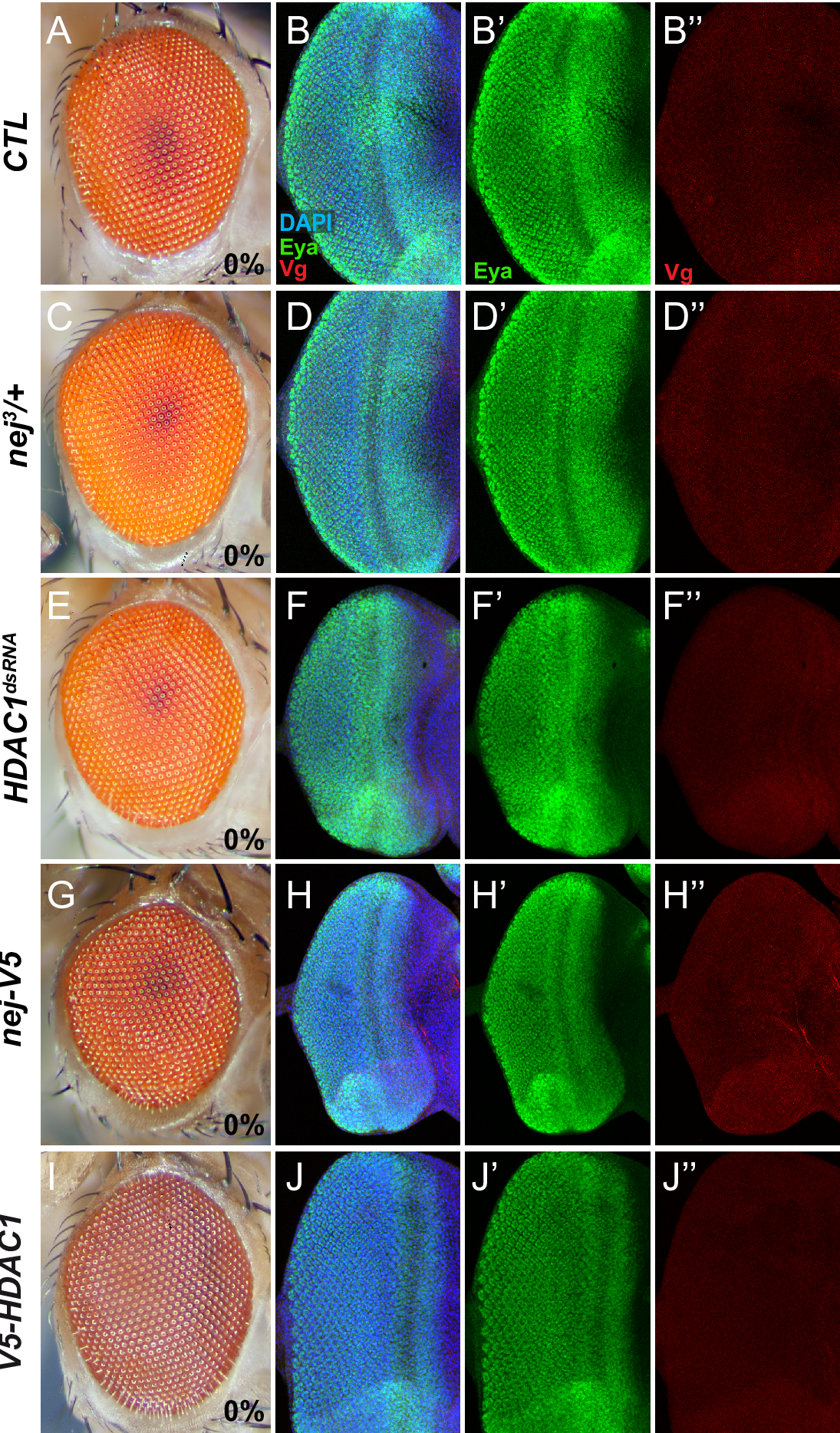

Supplement: S10 Fig — (A, C, E, G, I) Micrographs of adult Drosophila eyes. (B, D, F, H, J) Third instar larval eye discs immunostained with anti-Vestigial (Vg) and anti-Eyes Absent (Eya) antibodies. DAPI staining marks the nuclei. The following genotypes were analyzed: (A, B) ey-Gal4, UAS-GFP/+ referred to as CTL. (C, D) nej3/+; ey-Gal4, UAS-GFP/+. (E, F) ey-Gal4, UAS-GFP/UAS-HDAC1dsRNA. (G, H) ey-Gal4, UAS-GFP/UAS-nej-V5. (I, J) ey-Gal4, UAS-GFP/UAS-V5-HDAC1. Proportion (%) of eyes exhibiting ectopic wing material is indicated at the bottom right of each eye micrograph. Quantifications are shown in Table 2. (TIF) [file pgen.1009730.s010.tif]

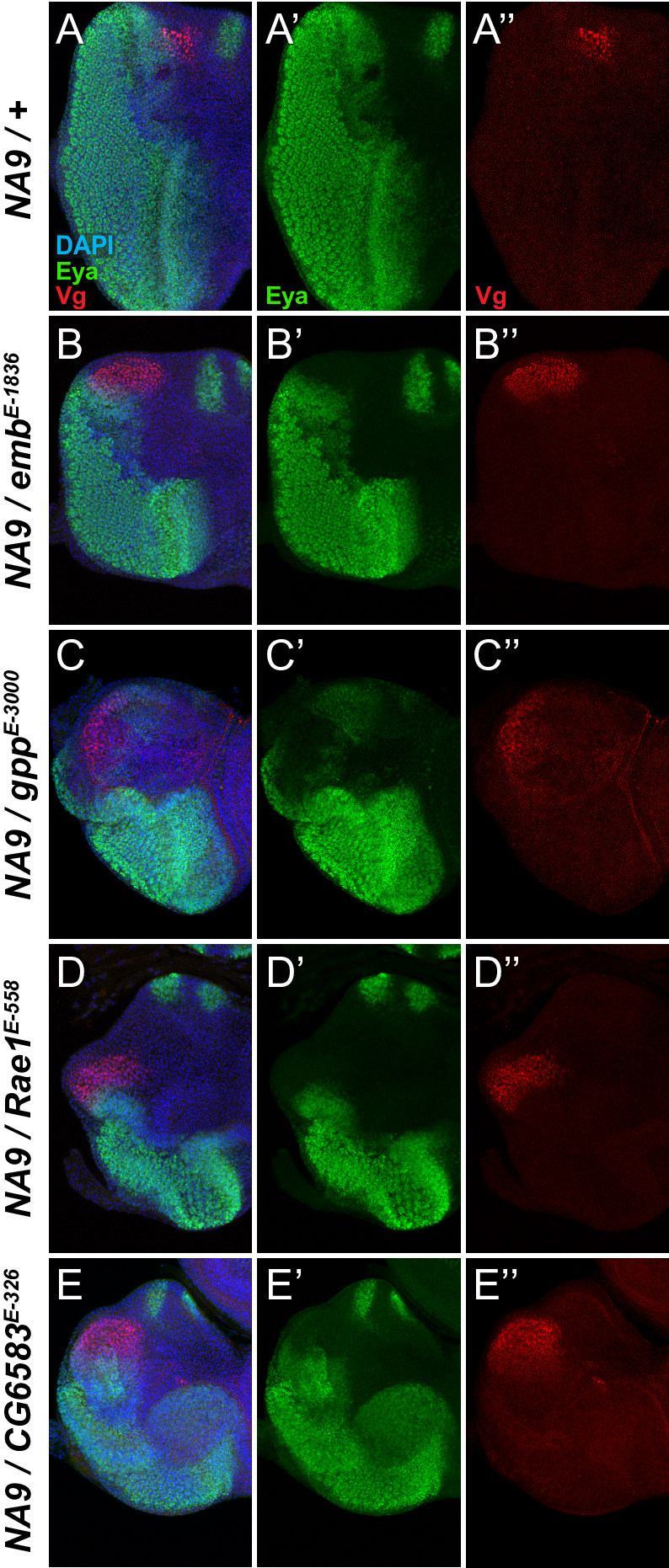

Supplement: S11 Fig — (A-E) Third instar larval eye discs immunostained with anti-Vestigial (Vg) and anti-Eyes Absent (Eya) antibodies. DAPI staining marks the nuclei. The analyzed genotypes are indicated to the left of the panels. (TIF) [file pgen.1009730.s011.tif]
