# Supplementary material for: A genetic screen in Drosophila uncovers the multifaceted properties of the NUP98-HOXA9 oncogene
Source: PLoS Genet. 2021 Aug 12;17(8):e1009730. doi: 10.1371/journal.pgen.1009730 (PMC8384169; doi:10.1371/journal.pgen.1009730)
Supplement: Attachment — Submitted filename: Response to reviewers.docx [file pgen.1009730.s015.docx]

**Response to reviewers**

***Reviewer #1:***

*The manuscript by Gavory and colleagues describes their efforts to use the Drosophila eye as a model to study acute myeloid leukemia (AML), a devastating human disorder. One inducer of AML is a translocation that fuses the N-terminal Phe-GLy repeats of NUP98 with the HOX9 transcription factor (ortholog of AbdA/B). Expression of this fusion (called NA9) in the Drosophila eye results in a small roughened eye. In this background the authors conducted a enhancer/suppressor screen and identified 29 complementation groups that modify the rough eye phenotype. A fraction of these are known chromatin modifiers. The authors focus on one type of modification – the transdermination of the eye into a wing. This phenomenon has been previously reported to occur in disc fragmentation/transplantation experiments, compound mutants in which Pax6 is down-regulated with other transcription factors, when the heterochromatin promoting gene winged-eye is over-expressed, and when Polycomb group membrs are downregulated.

The results in this paper are exciting for several reason and are appropriate for publication in PLoS Genetics. The authors were able to identify genes that are known to interact with NA9 in mammals thus validating the use of Drosophila as a model system. This, in and of itself, makes this study quite impactful. Second, the authors have uncovered a novel path towards transdetermining the eye into a wing. It appears that strong activation of Hox9/AbdAB targets along with modifications of epigenetic modifying factors are sufficient to switch the eye into a wing.*

*The paper is written very well, the figures are of the highest quality, and the data supports the conclusions. I would also say that the paper is very comprehensive. I only have few suggestions but overall I really like this manuscript.*

We thank the Reviewer for the kind and supportive comments.

***1.*** *I might have missed it but I think it would be a good control for the authors to deplete Vg and Scalloped (independently) and show that their loss suppresses the eye to wing transdetermination event.*

We agree with the reviewer that this would have been a good control to show. Unfortunately, we could not achieve it for technical reasons. First, we ordered *UAS* *dsRNA* lines targeting *vg* and *sd* transcripts from the Bloomington stock center, but only the *sd* *dsRNA* line led to endogenous Sd depletion (not shown). However, no effect was detected when the *UAS-sd^dsRNA^* line was driven by *ey-Gal4* in the presence of *UAS-NA9*. The reason relates to the fact that the *ey-Gal4* driver is silent in the NA9-induced transdetermined zone that has adopted the wing fate (i.e., the *ey* expression system is not active in wing cells). To circumvent this limitation, we tested the ability of *vg* and *sd* loss-of-function alleles to dominantly suppress the eye-to-wing transdetermination phenotype by introducing them as heterozygotes in the *ey-Gal4; UAS-NA9* background. However, no effect was detected when such crosses were conducted (not shown). Given the key role that Vg and Sd play in wing development, we conclude that their partial reduction caused by the heterozygous alleles is not sufficient to suppress NA9-induced transdetermination.

While we could not demonstrate the ability of Vg or Sd depletion to suppress the eye-to-wing transdetermination induced by NA9 expression, we would argue that the mere expression of the two wing markers (Vg and Sd) and the appearance of wing-like structures induced by NA9 expression remain compelling evidence that a transdetermination event has occurred.

***2.*** *It is interesting that depleting genes involved in activation of gene expression (nej) and repression of gene expression (E(Pc)) both lead to the eye to wing swith. Can the others comment further on this in the discussion.*

This is indeed interesting and as suggested by the reviewer, we added the following comment on this point in the Discussion section at line 487. Three new references were also included.

“E(Pc) was originally identified as an atypical member of the repressive Polycomb group (PcG) proteins because its loss-of-function did not lead to homeotic transformation as normally observed for other PcG factors, but it nevertheless enhanced PcG mutations (68). It was therefore considered early on as a transcriptional repressor. On this basis, it is intriguing that mutations in *nej*, which encodes a general transcriptional activator, behaved similarly to *E(Pc)* alleles and enhance NA9 activity. The function of E(Pc) has proven to be more complex than originally thought. Indeed, E(Pc) and homologous proteins in other species have been shown to be part of the Tip60/NuA4 multi-subunit complex that, in a context-dependent manner, act either as an activator or repressor of transcription, primarily through its HAT activity towards histones H2A and H4 as well as towards specific nuclear factors (55, 69, 70). Therefore, it may well be that in Drosophila, E(Pc), through the Tip60/NuA4 complex, and Nej control a set of functionally related genes that are critical for eye fate commitment and that their dysregulation upon *E(Pc)* or *nej* loss-of-functions enhances the eye-to-wing transdetermination event induced by NA9 expression.”

***3.*** *Could the authors conduct a small mini-screen where they over-express fly AbdA oir AbdB and deplete (with RNAi lines) some of the factors that were identified in their major screen to see if they can induce the eye to wing transformation. I think this would be very interesting no matter how it turns out.*

This would be an interesting experiment to conduct and we thank the reviewer for suggesting it. We did order available *UAS-AbdA* and *UAS-AbdB* transgenes from the Bloomington stock center and drove them during eye development using the *ey-Gal4* driver. However, the crosses led to fully penetrant mid-pupal lethality and therefore prevented us from assessing the impact on eye-to-wing transdetermination or testing for genetic interactions in adult flies. We also dissected eye-antennal discs from larvae to look for upregulated *vg*, *sd* and *wg* expression, but eye tissues failed to develop upon *Abd-A* or *B* expression leading to discs comprising only the antennal part (not shown).

***Reviewer #2:***

*Gavory and colleagues make good use of a genetic modifier screen in Drosophila to identify interactors of oncogenic NA9 fusion gene that is implicated in AML. Homologs of known modifiers of NA9 in leukemogenesis were identified, supporting the idea that modifiers found in Drosophila will apply to humans. The results suggest that NA9 perturbs several cellular functions. The manuscript is written well. The data are of good quality and most of the conclusions are supported by the data (exceptions described below). Appropriate controls are included such as those used to exclude the possibility that modifiers modify ey-GAL4 activity. The screen identified hth which was known to modify from direct testing, thus the data are internally consistent. The most interesting finding is that NA9 changed the fate of eye cells into wing disc cells. In principle, the questions being addressed here are suitable for publication in PLoS Genetics, but the analysis is mostly genetic interactions (although with various phenotypic outputs) and the reader is left with a gene list and not a mechanism. For example, the analysis that led to the finding that NA9 causes eye-to-wing transdetermination is superficial; the manuscript would be stronger if there was some indication of exactly how NA9 is doing this.*

We thank reviewer for the constructive comments regarding our manuscript.

*Specific comments are:*

***1.*** *The authors show that PIM and HD regions of NA9 are needed in fly, as they are for transformation in mammalian cells. But they have not shown that FG repeats of NUP98 are needed. In fact, HOX Delta NT without NUP98 reduced eye size on its own. Its effect is less pronounced than that of NA9 but is the difference statistically significant? In other words, what are the p values for the comparison between NA9 vs. PIM, HD, delta NT or delta CT mutants in Fig. 1H? Is the difference between NA9 and HOXA9 delta NT significant enough to support the conclusion that FG repeats matter? Otherwise, the conclusion that ‘these results indicate that the NA9 eye phenotype depends on the known functional elements of the oncoprotein’ is only partially supported by the data.*

The eye size difference between the NA9 and delta NT constructs is statistically significant. We now report the *p*-value (≤ 0.0014) as well as those for the other constructs as requested in the revised Fig. 1H. We have also added a new Supplementary Information file (Numerical Data & Statistics) that includes the raw data and statistics.

It should be noted, as reported in the original version of the manuscript, that the WT NA9 construct produces a fully penetrant expansion of the anterior dorsal head cuticle (Fig. 1C), while this is never observed with the delta NT construct (Fig. 1D).

***2.*** *The rescue of the NA9 eye phenotype by Exd and hth RNAi in Fig. 1 is very nice. But I am having trouble seeing the synergistic effect of hth and NA9 on tissue overgrowth in Fig 2. Is overgrowth measured from adult eyes or eye discs? If the adult eye, is overgrowth being measured beyond the expected adult eye size and how? If in eye discs, is it the GFP area that is measured? If so, I cannot see that NA9 + hth in Fig. 2H shows overgrowth over hth or NA9 alone. If anything, there seems to be less GFP in Fig. 2H than in Fig. 2F.*

We agree that this point needs to be clarified.

Tissue overgrowth was measured in adult eyes only and is reported in Fig. 2I (lower bar graph). Measurements were conducted by outlining the overgrowths using the Lasso tool in Photoshop. The areas (square pixels) were then determined using the Measurement log. The average size of tissue overgrowths produced by NA9 and HTH co-expression was normalized to 1 and used for comparison to other genotypes. The area value was set to 0 when no overgrowth was detected. Eye size (Fig. 2I, upper bar graph) was measured in a similar manner, but in this case, the average size of control adult eyes (*UAS-LacZ*) was normalized to 1. This information has now been added to the revised Materials and Methods section. A new Supplementary Information file (Numerical Data & Statistics) reports the raw data and statistics.

Furthermore, for greater clarity, we modified the text in the Results section as follows:

**Original sentence (line 226):** “These results demonstrate the ability of NA9 and HTH to cooperate in an EXD-dependent manner.”

**Revised sentence (line 226):** “The emergence of cuticular overgrowths in adult eyes when NA9 and HTH are co-expressed suggests a cooperative event, reminiscent of the functional collaboration observed between NA9 and MEIS1 in mammalian systems.”

***3.*** *The authors identified a previously undocumented activity of NA9 in transdetermination, from eye to wing in this case. They also identified a number of genetic/epigenetic modifiers of this effect. These are very interesting data. But an obvious connection is not being made. A large body of literature on transdetermination in Drosophila implicates Wg as playing a central role and some of the work is cited by the authors. The authors see up-regulation of Wg when they overexpress NA9, and this effect is greater in the dorsal part, which is where they see transdetermination (Fig. S1). Therefore, they should test if Wg is needed for NA9-induced transdetermination. Conditional Wg alleles or conditional expression of inhibitors may be used. In any case, some insight into the mechanism of transdetermination would improve the manuscript.*

Unfortunately, our attempts to demonstrate a role for Wg in NA9-induced eye-to-wing transdetermination were inconclusive. As explained above (point 1, reviewer 1), the *ey-Gal4* driver is inactive in the NA9-induced transdetermined zone due to the eye-to-wing fate change and thus Wg (or any other factor) cannot be depleted by RNAi in this area when NA9 is co-expressed. To circumvent this limitation, we tested the ability of several *wg* heterozygous alleles to suppress the NA9 eye phenotype. However, none of these altered the phenotype, which is consistent with the fact that no *wg* alleles have been recovered in our NA9-dependent genetic screen. These results suggest that Wg is not required for the NA9-induced eye-to-wing transdetermination or that halving *wg* gene dosage is not sufficient to modify the *NA9*-induced eye-to-wing phenotype.
